# Teaching without boundaries: interviews exploring the adaptation of collaborative inquiry to the American context

**DOI:** 10.12688/gatesopenres.14168.1

**Published:** 2023-05-15

**Authors:** Olivia G. Carr, Xiu Cravens

**Affiliations:** 1 Department of Leadership, Policy, and Organizations, Vanderbilt University, Nashville, TN, 37203, USA; 2 Education Resource Strategies, Watertown, MA, 02472, USA

**Keywords:** collaboration, teachers, inquiry, policy adoption, TPEG, lesson study

## Abstract

Purpose: This study qualitatively examines the efforts of implementing teacher-led collaborative inquiry in American public schools to improve instruction. We focus on a model called Teacher Peer Excellence Group (TPEG), designed to capture the essence of Japanese lesson study and Chinese teaching-study groups that involve lesson planning, peer observations, feedback, and revision.

Methods: We conduct qualitative case studies in three pilot schools using a constructivist research paradigm.

Findings: We identify action steps essential to introducing and sustaining the TPEG model and pathways to local adaptation.

Implications for research and practice: The study contributes to the body of research that seeks to understand the role of instructional leadership and teacher decision-making in successful school-level initiatives.

Highlights

- Described pathways of local adaptation towards building the necessary structure for disciplined collaborative inquiry.
- Identified action steps related to instructional leadership central to the implementation and sustainability of school-level initiatives.
- Reviewed the essential objectives of building a professional knowledge base for teaching.

## Introduction

The past few decades have revealed growing interests in fostering teacher collaboration to improve instruction and student learning ( Achinstein, 2002; Bond, 2014; Bruce *et al.*, 2016; Goddard *et al.*, 2007). However, too often, collaboration initiatives fail to take root in the day-to-day operation of schools ( Giles & Hargreaves, 2006; Sindelar *et al.*, 2006; Zech *et al.*, 2000). This phenomenon has prompted questions about the necessary organizational conditions and decision-making processes for successful local adoption and adaptation of educational reforms over time.

The purpose of this study is to provide insight into the efforts of implementing teacher-led collaborative inquiry – defined as teachers engaging in consistent and critical inquiry of their teaching practice ( Butler & Schnellert, 2012; Morris & Hiebert, 2011) – as a driving mechanism for instructional improvement in American public schools. We aim to capture the nuances in teaching culture and organizational structure, identify the action steps essential to introducing and sustaining school-level initiatives, understand the role of instructional leadership, and explore how variations in decision-making influence local adoption and adaptation.

We focus on a model called Teacher Peer Excellence Group (TPEG), which was intentionally designed to capture the essence of the Japanese lesson study and Chinese teaching-study groups ( Fujii, 2016; Huang & Shimizu, 2016; Jensen *et al.*; Lewis *et al.*, 2006), and modified for the American educational context ( Cravens & Drake, 2017). Teachers in this model lead subject-specific collaborative inquiry cycles. Each cycle involves lesson planning, peer observation, feedback, and revision of lesson plans. Drawn from prior literature on situated learning and communities of practice ( Hiebert *et al.*, 2002; Wenger & Snyder, 2000), the TPEG model aims to build a professional knowledge base for teachers that has three key signposts: (1) deprivatized practice, (2) storable and shareable teaching materials, and (3) a mechanism for verification and improvement.

We explore the fertile ground for follow-up research where, five years after the initial implementation of TPEGs in 27 schools from six districts in Tennessee, the pilot schools have taken different paths in how they integrate the model into the existing organizational structures and routines. Specifically, we conduct case studies in three schools that have adopted the TPEG model to varying extents in different settings. We ask two research questions: (1) What action steps were taken by schools to adopt and sustain collaborative inquiry cycles? (2) Compared to the theory of change of how TPEG was intended to work, what local adaptations were made to the TPEG model and why?

Using qualitative analysis, we identify five action steps related to instructional leadership that were central to the implementation and sustainability of collaborative inquiry in these schools: forming collaborative teams; scheduling collaborative time; learning to collaborate; setting expectations for collaboration, and cultivating buy-in. We also describe how teachers and school administrators interpreted and adapted each aspect of the collaborative inquiry process, with particular emphasis on if and how they align with the theoretical framing of collaborative inquiry.

## From collaborative inquiry to instructional improvement

### Collaborative inquiry cycles

Studies on teacher practice point to teacher-led collaborative inquiry as a promising form of in-service professional development ( Akiba & Wilkinson, 2016; Cravens *et al.*, 2017; Cravens & Hunter, 2021; Goddard *et al.*, 2007; Huang & Shimizu, 2016; Lewis, 2015; Saunders *et al.*, 2009). The conceptualization of collaborative inquiry is grounded in the socio-constructivist model of self-regulated learning ( Butler & Cartier, 2004; Butler & Schnellert, 2012) and situated learning theory ( Wenger *et al.*, 2002). Applied to teaching, prior research suggests that self-regulation and meaningful change occur when teachers engage in recursive cycles of goal-directed, job-embedded, ongoing, and critical inquiry of practice ( Butler & Schnellert, 2012; Bryk *et al.*, 2015; Gallimore *et al.*, 2009; Morris & Hiebert, 2011). In inquiry models, teacher teams are trained to participate in iterative cycles that involve setting instructional goals, lesson planning, implementing the lesson plan, observing peers teaching, and monitoring learning results. Furthermore, subsequent cycles of collaborative inquiry are informed by findings from previous cycles.

Collaborative inquiry on instructional practice occurs mostly outside the United States. The Japanese lesson study model was one of the first to be introduced to the United States in the 1990s ( Hiebert *et al.*, 1999) as a model of action research that facilitated teacher enactment of ambitious instruction with the potential to scale up effective teaching aligned with external standards ( Hiebert *et al.*, 2002; Lewis *et al.*, 2006; Lewis, 2015). Studies have also associated the “teaching-study groups” in Shanghai with high student achievement while maintaining a low correlation between socioeconomic status and academic proficiency ( Jensen *et al.*, 2016; OECD, 2011; Tucker, 2014; Wang, 2013; Yang, 2008).

**Figure 1.**
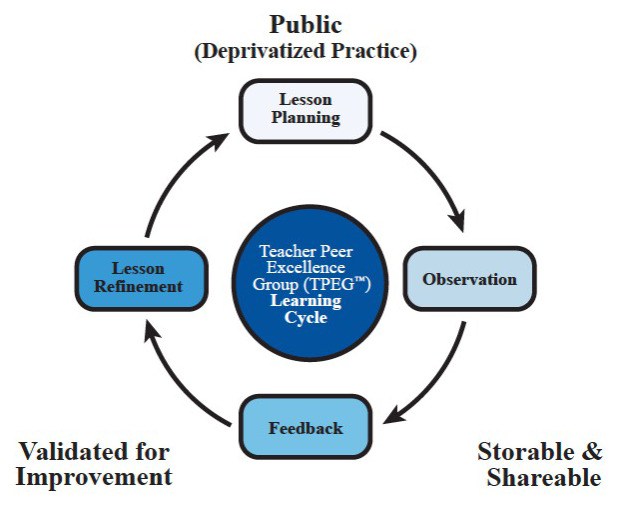
the TPEG collaborative inquiry cycle.

The theory of change for the collaborative inquiry model (see Figure 1) highlights three requirements to transform what teachers gain from day-to-day practice to a professional knowledge base ( Hiebert *et al.*, 2002; Stigler & Hiebert, 2016): (1) Teachers make their practice public through collaborative lesson planning, peer observations, and peer feedback; (2) the materials and expertise gathered during inquiry cycles are cumulative, accessible, and shareable to other teachers so that teachers do not have to “reinvent the wheel” for each new teaching assignment; (3) there is a mechanism for validating improvement by experts and peers. To reach these objectives, the teaching-study groups China are typically organized by subject and grade level, led by teachers with content and pedagogical expertise. Teams then engage in weekly inquiry cycles of lesson planning, peer observation, feedback, and lesson revision ( Cravens & Wang, 2017; Wang, 2013).

### Enabling conditions for professional development

Prior research on in-service professional development highlights several vital organizational conditions that are associated with success and sustainability: instructional leadership, professional community, trust, and teacher efficacy.

***Instructional leadership.*** Leaders are key stakeholders who affect student learning by making numerous daily decisions. They influence the school organization and the people within it, including (re)designing the structure of the school, shaping expectations and school culture, developing teachers’ pedagogical and content knowledge, and cultivating professional communities ( Coburn, 2001; Goldring *et al.*, 2009; Leithwood *et al.*, 2004; Printy, 2008; ten Bruggencate *et al.*, 2012; Youngs & King, 2002). Principals who focus specifically on improving classroom practices are considered ‘instructional leaders’ ( Hallinger & Heck, 2010). Instructional leaders can use their influence to shape structural factors, such as time, that are necessary to support collaborative inquiry cycles and direct teacher efforts toward student learning. Using a meta-analysis, Robinson *et al.* (2008) find that instructional leadership has a stronger influence on student outcomes than do other types of leadership, such as transformational leadership, for which leaders focus on inspiring staff to better engage with their work.

***Professional community.*** A schoolwide professional community consists of teachers who frequently interact using a set of shared norms about improving teaching and learning. More specifically, teachers in professional communities reflect on instructional practices and student learning, observe each other’s teaching practices, problem solve together, and share work through peer collaboration ( Bryk *et al.*, 1999). In a study of 24 schools, Louis *et al.* (1996) find that professional communities have a positive relationship to teachers taking responsibility for student learning, and Louis and Marks (1998) report that professional communities positively affect classroom organization and student academic performance.

***Trust.*** Bryk and Schneider (2002) and Louis (2006) argue that trust within schools is essential to facilitate daily practice and improvement measures. They write that trust between faculty members in a school is built on respect, competence, a personal regard for other people, integrity, and agreement on issues such as what students should learn and how teachers should instruct and behave. Effective principals can improve their schools by building trust between their teachers ( Hoy & Tschannen-Moran, 2007; Youngs & King, 2002), or teachers can develop trust themselves over time in a way that allows them to work together well and take full advantage of the benefits that can come from collaborative inquiry cycles.

***Teacher efficacy.*** Teachers who believe they can positively affect student learning are more likely to engage in collaboration for instructional improvement ( Bruce & Ross, 2008; Tschannen-Moran & Hoy, 2001). Pedagogical efficacy means that teachers feel they can successfully integrate new practices, like collaborative inquiry cycles, into their regular practice ( Bandura *et al.*, 1999). Bandura *et al.* (1999) asserts that teachers use four types of information to shape their efficacy: (a) mastery experience – the perception that their teaching has been successful through their own experiences; (b) vicarious experiences – teaching experiences successfully (or unsuccessfully) modeled by someone else; (c) social persuasion – encouragement and/or specific feedback to teachers about their teaching; (d) affective states – anxiety or excitement related to teaching, perhaps from receiving results on a recent standardized test. Collective efficacy, therefore, is achieved when a group of teachers believe that they have the power to affect and teach students. Goddard *et al.* (2000) add that teachers analyze the teaching task and assess their collective teaching competence to shape whether they think they will be successful. Collective teacher efficacy leads to teachers more purposefully working to pursue common goals and enhance student learning ( Goddard *et al.*, 2000).

### Challenges to implementation

Prior research finds that existing structural and cultural norms in schools can make or break the introduction of change to teacher practices ( Coburn, 2005; Huang & Shimizu, 2016; Jensen *et al.*, 2016; Rose, 1991). Too often implementers of new models fail to garner the buy-in of stakeholders, contextualize imported practices, or weigh tradeoffs in adaptation ( Akiba & Wilkinson, 2016; McDonald, 2012).

Taking the cognitive approach to study reform implementation, researchers argue that school administrators and teachers draw on their own knowledge to interpret and translate imported approaches and are likely to make modifications and create incremental change – a sensemaking process ( Coburn, 2005; Spillane *et al.*, 2002). For example, Roehrig *et al.* (2007) use mixed methods to examine 27 high school chemistry teachers as they implemented a new curriculum. They report that teachers’ beliefs and preferences for their teaching and the presence or lack of a supportive network within their schools had a strong influence on implementation. In particular, teachers who primarily used inquiry-based teaching made the transition to the new curriculum more smoothly than teachers who primarily used traditional teaching methods (with instructor-directed lessons that focused on lectures and worksheets), and the most effective support for the new curriculum came from school administrators when they met with teachers to discuss student learning.

Groves *et al.* (2016) examine the lesson study model in three Australian schools. The collaborative teams were tasked with implementing structured problem-solving lessons, and they found success in deep lesson planning, allowing large numbers of participants to observe their classes, and insight from the “knowledgeable other.” However, the teachers had difficulty matching the Japanese problem solving lesson structure with the prescribed Australian curriculum and mirroring the Japanese model because of the Australian teaching culture that emphasizes small group instruction rather than whole class teaching.

Studies also show that stakeholders’ sensemaking about change is influenced by the conditions and organizational structure in their schools ( Ketelaar *et al.*, 2012; Ng & Tan. 2009). For example, Akiba and Wilkinson (2016) use extensive mixed method research to describe how the implementation of the lesson study model was limited to shortened and simplified versions in Florida. They find that implementation was hampered by the lack of systemic capacity building for key stakeholders to understand the importance of integrating the new model with the existing organizational structures and routines of teacher professional development.

Intentional and continuous local adaptation is also necessary. McLaughlin (1987) describes implementation as “a process of bargaining or negotiation” (pg. 175), with policies adapting to the local context and the site adapting to the reform, sometimes called mutual adaptation. Adoption of a new practice should therefore include “additional, individual teacher-directed design, fitting, and adaptation for local circumstances” ( Barab & Luehmann, 2003, p. 464), while still maintaining the integrity of the reform. As an example, Spillane (1999) examines the responses of nine local education agencies (LEAs) to state standards reforms and find that the LEAs adopted the new standards easily but overly adapted the more complex and newer characteristics of the reforms, which led to procedural compliance instead of substantive compliance and change. Local adaptation without losing substance is therefore important to the success and sustainability of new practices.

## Teacher peer excellence group (TPEG)

In 2013, researchers from American and Chinese universities designed the TPEG model based on the principles of the Japanese lesson study and Shanghai teaching-study groups ( Jensen *et al.*, 2016; Lewis *et al.*, 2006; Wang, 2013) with flexibility for local adaptation. There are four steps to each cycle:

(1) Lesson planning: The TPEG chooses the particular concept or lesson to cover. The teachers pull from resources (including, preferably, a shared repository of lessons) and their own expertise and experiences to plan the lesson collaboratively.
(2) Observation: One or two teachers then teach the lesson for others to observe. Importantly, the teachers are observing to evaluate the lesson, not the teacher. Ideally, a content and pedagogical focus has been identified through lesson planning, and the observers use an agreed-upon rubric that measures instructional quality.
(3) Feedback: Peer feedback focuses specifically on the targeted instructional objectives, the successes and challenges of the lesson, and how to best improve on these.
(4) Revision: The feedback session directly feeds into improving the lesson for future use. If every teacher in the TPEG has not yet taught the lesson, the remaining teachers will teach the revised lesson. This might trigger another round of feedback and revision of the lesson. After multiple trials, teachers then store the lesson and accompanying notes in a way that is accessible by other teachers and in future years.

The TPEG collaboration model was piloted in volunteer schools across six districts (18 schools) in Tennessee in the 2013–2014 school year, and nine additional schools were added for the 2014–2015 year. Principals and teachers in pilot schools received training on TPEGs, protocols for conducting meetings, and a template to plan and document inquiry cycles. To start, principals worked with the research team to identify two TPEGs in each school, preferably organized by subject matter or grade level. The principals then spent a week in Shanghai observing and discussing the local version of the teaching-study groups in a wide variety of schools. Upon their return, principals were encouraged to work with their TPEGs and develop collaborative inquiry cycles to best fit the structure and needs of their own schools.

The research team intentionally designed the implementation of TPEGs to be flexible in how each pilot school would conduct their inquiry cycles, as long the cycles were ongoing, completed with the four key steps, and used the state teacher evaluation rubric as the inquiry focus. During the first two years, the collaborative inquiry cycles varied from one to six weeks in the pilot schools. This loose-tight design, with flexibility in all but a few very important aspects, was chosen to provide sufficient protocol and discipline to the collaborative inquiry while ensuring that it was adaptive to local conditions and needs. Principals and TPEG teachers took the lead in deciding team formations and the logistics for inquiry cycles.

Grant funded technical support for the pilot schools ended in the summer of 2015. Since then, follow-ups by the research team have shown that there had been large variations in how pilot schools engaged in these disciplined collaborative inquiry cycles. For the TPEG model to have a more significant impact on instructional improvement, an in-depth exploration was needed to understand how schools addressed the challenges of structure, culture, and resources to implement the TPEG model. This study seeks to fill that gap by providing a formal examination into these issues.

## Methods

### Study settings

We used purposive sampling to select three schools that had various levels of success in implementing and sustaining collaborative inquiry cycles as prescribed by the TPEG model. These schools also vary by urbanicity, size, and demographics, as shown in Table 1. These three schools were sufficiently different to provide a range of experiences with and insights into the TPEG pilot, so no further schools were necessary to recruit.

**Table 1.** School characteristics for sample schools.

	Elwood Elementary School	Granville Elementary School	Clark Middle School
**Urbanicity**	Urban	Rural	Rural
**Grades**	Kindergarten to 5 ^th^ grade	Prekindergarten to 4 ^th^ grade	5 ^th^ to 8 ^th^ grade
**Approximate # teachers**	50 teachers	20 teachers	35 teachers
**Approximate # students**	1000 students	400 students	800 students
**Approximate student ** **demographics**	85% white, 10% economically disadvantaged	90% white, 30% economically disadvantaged	90% white, 20% economically disadvantaged
**Teacher plan time**	45 minutes per day	50 minutes per day	90 minutes per day
**Mandatory collaborative ** **time for lesson planning**	N/A	25 minutes 4x per week	45 minutes per day

Sources: State Report Card (n.d.) and interviews

All three schools are located in Tennessee, where the average student test scores on the National Assessment of Educational Progress are average or below average compared to those of other states but are improving quickly over time ( SCORE, 2019). ^1^ Tennessee has also implemented several state initiatives since 2013 that seek to improve teacher performance through stronger in-service development, including one relevant for this study, the Instructional Partnership Initiative (IPI). IPI is a voluntary program that pairs teachers based on their strengths and weaknesses in their teacher evaluations and/or principal recommendations so teachers can learn from each other ( TDOE, 2017). The concurrent implementation of an initiative with similar objectives but different approaches from the TPEG model added to the complexity of local implementation.

The first school, Elwood Elementary, is in an urban county, and it was recognized by the state prior to this study for improvements in student achievement, closing achievement gaps, and teacher value added. Starting in the 2013–2014 school year, two grade-level TPEGs were added each year until every grade level had a TPEG. These teams met weekly to complete inquiry cycles lasting two weeks. For five years, official TPEG cycles occurred at various frequencies each year, though TPEG cycles were abandoned during data collection for this study, due to an increase in discipline problems at the school.

The second school, Granville Elementary School, in a different rural County, did not sustain the TPEGs in their original form but had success in modifying the model. The school piloted the TPEGs starting in 2014-2015 with two teams that were vertically aligned, meaning teams spanned grade levels. These teams did TPEG cycles once per semester for two years before the school switched to a collaborative practice that evolved out of TPEG. Granville Elementary had required collaboration, and teachers also participated in IPI, the state peer observation initiative. While IPI observations were not connected to specific lessons, as they were with TPEG, the two models share certain characteristics of collaborative inquiry. It is therefore informative to examine why this observation-focused model has been sustained at Granville for several years, whereas collaborative inquiry observations have not.

The third school, Clark Middle School, fully implemented collaborative planning schoolwide. It is of note that Granville Elementary School and Clark Middle School are within the same county and therefore under the same school system. Spearheaded by an assistant principal, the school started the TPEG model with two subject-specific teams in 2013–2014. In the second year, the principal decided to expand TPEG so it could benefit more students at once. The school leadership team restructured the master schedule to allow for 45 minutes of mandatory collaborative planning time every day, though teachers ceased peer observations. The school frequently hosts educators from across the state who observe and ask questions about their collaborative practices.

### Data Collection and Analysis

We employed a case-study design to qualitatively examine these complex and dynamic school settings. Data collection took place from the spring of 2018 to the fall of 2019. After pilot interviews, interviews and observations were conducted in-person at Granville and Clark and by phone ^2^ at Elwood. A convenience sample of teachers were selected based on their availability before school, after school, or during planning periods. This allowed for some stratification by grade, as each team had common plan times that were used for observations and interviews. Interviews were recorded and lasted from four to 55 minutes, and observations were approximately 30 minutes each. Respondents who participated in shorter interviews seemed comfortable speaking to the interviewer and responded in similar ways to their peers. At Elwood, four teachers declined to be interviewed, though more did not answer a recruitment email. At Granville, two teachers declined to be interviewed, both because they were too busy at the time, and at Clark, only one teacher declined to be interviewed. Overall, we have 48 interviews and 15 observations of collaborative sessions, as described in Table 2.

**Table 2.** statistics on the qualitative data collected.

	Elwood Elementary School	Granville Elementary School	Clark Middle School
**# Principals interviewed**	0	2 (100% of total)	3 (100% of total)
**# Major subject teachers interviewed**	7 (~15%)	10 (~50%)	23 (~75%)
**# Special area teachers interviewed**	0	2 (~50%)	1 (~10%)
**Median experience in the education** ** profession of participants**	10 years	15 years	14 years
**Average interview length**	40 minutes	13 minutes	12 minutes
**# of observations of collaborative sessions**	0	5	10

A semi-structured interview protocol (see the extended research materials here: Carr & Cravens, 2022) was used to ask teachers about collaboration and professional development, particularly the strengths and weaknesses of their collaborative inquiry experiences. We first used skip patterns, which adjust the interview protocol to include only relevant questions, to ask the teachers about their background and experience with collaborative inquiry. We followed with interview questions that aimed to capture their perceptions of TPEG implementation and changes to teachers’ collaborative practices. For instance, we asked teachers to describe a typical collaborative cycle – including who attended collaborative meetings and how the group made decisions – both when TPEG was first introduced and as related to their current practices. The questions also addressed ideal collaborative environments and professional learning opportunities. There was a separate interview guide for principals and instructional coaches that had the same structure but included more specific questions regarding the role of various key stakeholders in decision making around adapting the cycles over time.

The interviewer used the interview protocols as a loose guide for the conversation, in particular by altering the order of questions frequently to help respondents flow from one topic to the next naturally. Between interviews, the researchers updated the interview protocol as needed, primarily to get more specific information on current non-TPEG collaborative practices. For the observations, the researcher attended collaborative team planning sessions for approximately 30 minutes each and focused on time use and the type and quality of interactions between the participants on each collaborative team.

The first author collected all data for this study. As a young, white, educated female who lived in Tennessee, she resembled a typical teacher in the pilot schools, and all participants seemed comfortable and glad to share their experiences. Collaborative teams appeared to use their collaborative time as usual, particularly after being told that the observations focused on the structure of their time and interactions, rather than performance. After the interviews were transcribed by the first author or a transcription service, analyses were conducted using the software Dedoose using open and hierarchical coding to identify details that were salient to participants inductively and deductive coding based on the TPEG conceptual framework. This included identifying enabling conditions that are named in the literature review, how to promote buy-in, other initiatives, and support for collaborative inquiry that allowed it to be continually successful for some teachers and not others. Each step of the TPEG cycle and how it adapted over time were examined next, followed by teacher instructional practices and instructional improvement.

### Ethical approval

This study, including all recruitment materials, consent forms, and data storage and privacy decisions, was approved by the Institutional Review Board at Vanderbilt University. Required permissions were given at the district-level and by the school principals. Participants each signed an IRB-approved informed consent form prior to interviews. Participants received a $20 gift card as a thanks for their participation.

## Findings

### What are the key action steps that schools used to adopt and sustain collaborative inquiry cycles?

Our interview data indicate that school leaders created collaborative environments by promoting collaboration to teachers as a positive change, making it mandatory, and supporting teachers while they found ways to make the change work. While the actual processes were more nuanced, lessons from these schools shed light on specific steps to address challenges in organizational structure and teacher sensemaking. Table 3 summarizes the major findings, which are elaborated below.

**Table 3.** Summary of findings of key action steps for collaborative inquiry cycles.

Action steps	Summary
Forming collaborative teams	Sustained collaborative teams consisted of 2-4 grade-subject matched teachers.
Scheduling collaboration time	Sustained collaborative time was common time embedded in the school day that would occur daily or almost daily.
Learning to collaborate	A difficult part of collaborative inquiry was learning how to collaborate productively, particularly with giving and receiving constructive feedback.
Setting expectations for collaboration	Schools and collaborative teams with high expectations to participate in collaboration and create quality lessons were more successful in sustaining collaboration.
Cultivating buy-in	Principals bought into the TPEG model after seeing it work well and flexibly in Shanghai schools. Teachers bought into the TPEG model after seeing it improve their own instruction and/or time management.

Forming collaborative teams

When TPEG was introduced, the first necessary decision was how to group teachers into collaborative teams. Prior literature on lesson study and teaching-study groups underscore the importance for members of the learning community to have shared interests in solving “problems of practice” that are specific to a focal subject area ( Hiebert *et al.*, 2002; Wang, 2013). Studies also find that teachers can learn more about how their practice affects student learning when they focus on a specific teaching or learning issue over a period of time and when teachers repeatedly experiment with different instructional strategies in similar and different settings ( Gallimore *et al.*, 2009). We find that the actual formation of the TPEGs in the pilot schools varied by grade level, school size, and focal subject. As examples, because there were only one or two teachers per grade, Elwood Elementary formed math and reading TPEGs that spanned multiple grades. Teachers at the larger Clark Middle School designated subject-grade teams for math and reading and either subject-specific teams that spanned multiple grades or grade-specific teams that spanned two subjects for the smaller number of teachers who taught science and social studies.

While such variations in TPEG formation were largely due to differences in school size and grade structure, teachers confirmed in interviews the advantages of forming collaborative teams by subject and grade whenever possible. They pointed out that the benefits of “vertical” alignment were often overshadowed by time constraints and student needs from different grades. One Clark Middle School teacher gave an example of why vertical teams did not work well:

“For example, the first [TPEG] one we did was a 5 ^th^ grade English lesson. It didn’t seem all that applicable to many of us, because they were focusing so much on fluency and basic comprehension and parts of their standards that we don’t even have those sorts of standards in [grades] 6, 7, 8. So I remember that that was– You really felt like you were helping one other person’s lesson or that 5 ^th^ grade group’s lesson, but you didn’t feel like it necessarily applied to you.” – Teacher LC ^3^

While teachers in cross subject collaborative teams saw the value of diverse perspectives, many shared that they would have preferred to be with teachers who had shared content-specific expertise. We also find that while TPEGs tended to be large – about six people at Clark, up to eight at Elwood – the sustained collaborative teams were smaller with two to four people in each team. The size was not as salient as the composition of the collaborative teams to teachers and principals, however, so larger teams might be appropriate in schools with more teachers per grade or grade-subject.

Scheduling collaborative time

Identifying and setting up shared collaborative time was a major concern in all three schools. We find that sustained collaboration occurred during planning periods embedded in the school day that were made available for all teachers of a collaborative team. The success of this was most evident in Clark Middle. Its assistant principal discussed the tradeoffs inherent to ensuring that teachers had enough collaborative and individual plan time during the school day, namely that administrators were able to give their teachers 90 minutes of daily planning time by increasing class sizes and decreasing the amount of planning time for special area teachers.

Given time restrictions, it is also important to carefully determine the frequency of the collaborative inquiry meetings. TPEGs were asked to meet approximately once per week to focus on one particular lesson over the course of two weeks. One administrator explained why her school decided to shorten the inquiry cycles to one day: “The two week [TPEG] cycle…it took so long, we weren’t getting enough bang for our buck…We were able to arrange our schedule, it worked out where we could impact every single kid every day” (Assistant Principal PC).

Quick cycles allowed teachers to adjust their teaching quickly, but it also meant that teachers could not easily incorporate parts of the inquiry process into their cycles. Collaborative lesson planning also became part of the daily teaching practice, rather than a distinct activity that used research techniques to deliberately focus on creating and testing particularly high-quality lessons. Related, the short cycle length and common planning times did not allow for teachers on a collaborative team to easily observe each other teach.

Learning to collaborate

 Between 2013–2015, principals and TPEG leaders attended training sessions that demonstrated best practices in conducting planning sessions and providing constructive feedback with depth and reasoning. These administrators were tasked with explaining and modeling collaborative techniques at their home school and ensuring that their teachers were building a professional community and trust amongst themselves. At Clark Middle School, the leadership team did this by showing videos from her trip to Shanghai, modeling good collaborative practices in front of and with collaborative teams, sharing research on collaboration, and providing a checklist of how to productively collaborate. As the principal explained, “The very first thing is to review the lesson they’d just taught. What was good, what was bad, what needs to be changed, and then where do we need to go from here, and that’s when today’s lesson [planning] begins” (Principal OC).

Establishing Expectations for Collaboration

Our interviews show that principals must cultivate a culture of high expectations for collaboration to maintain fidelity and improve rewards from the collaboration, both of which increase teacher buy-in and ownership over the process. Clark Middle School is the perfect example of this because the principal maintained extremely high expectations for fidelity to his school’s collaboration routine when collaboration was first scaled up to the entire school, and that eventually resulted in a strong culture of productive collaboration. The principal explained,

“They had an hour and a half planning period. The first 45 minutes had to be co-planning, and there were no exceptions to that. Not going to the copy machine. Not having IEP meetings. Not going to get a snack out of the [vending machine] thing. This is co-planning time…If your expectation is that they will be doing this for 45 minutes, and if they’re in the hallway, that it is addressed very quickly and that there’s no doubt about what they’re supposed to be doing for those 45 minutes. And then if you do that a couple of times, everybody has [it].” – Administrator OC

At Granville Elementary, the expectations were not as strict, and the resulting collaborative practices were less cohesive. Elwood Elementary School is a contrasting case in that, at the time of data collection, some teams participated while others did not, as there was not a formal expectation that teams collaborate.

Teachers noted that mandatory collaboration gave them the necessary push to take the “extra” step of collaborating, and that while some teams found intrinsic reasons to sustain collaboration, there were many ways that collaboration could have been derailed. We found that giving teachers more flexibility might allow them to better shape their collaborative practices to their own needs, but if this were to happen, we would expect some teams to scale back collaboration and return to individual planning, as happened at Elwood Elementary.

Cultivating Buy-In

Trust, efficacy, and professional community were important in sustaining collaborative inquiry cycles, largely through establishing buy-in. Participant comments show that it took time to cultivate buy-in for collaboration. The assistant principal at Clark Middle School said that her trip to Shanghai was “vital” to prove to her that it would work, and it took about a year for her teachers to see the fruits of their labor (high-quality lessons stored in a central location) and be convinced that collaboration was a good idea. Principals in all three schools tried to cultivate interest, relate stories to their teachers, and roll out collaboration slowly to promote buy-in, but their eventual success came from teachers seeing improvements in their own practice and workload over time after administration made the collaborative practices mandatory. Only then could the principals turn the TPEG collaboration from an administration-led initiative to a teacher-led initiative.

Buy-in was also developed through the efforts and patience of the teachers themselves. The teachers at Clark Middle and Granville Elementary seemed to have strong shared norms about student learning being at the center of their practice and about their collective responsibility for all the children in their grade. Related, we noticed that teachers at Elwood Elementary who did not like collaboration spoke frequently about their preferred teaching styles, rather than about practices that would most help their students learn.

Many teachers identified personality clashes as the easiest way to disrupt collaborative relationships. In particular, personality clashes tended to happen when teachers on a collaborative team had very different teaching styles or when one teacher attempted to control the decision making in a way that was unwelcome. We saw that while mandatory collaboration urged teachers to learn to work together productively, sometimes an administrator or other neutral party with some authority, like an instructional coach, could mediate relationships. These authority figures would help members of the team align their goals and priorities and follow best practices.

Developing trust among peers was a prevalent theme from the teacher interviews. Teachers listed two reasons that trust is essential to sustaining collaborative inquiry: They could be vulnerable in front of their teammates to make their teaching public, and they had to rely on their peers to produce high-quality work. An assistant principal described the fears of some of her teachers:

“If I open my planning time to you, and you’re the other teacher coming in, and we’re going to plan together, what if your ideas aren’t as good as mine?…So it’s overcoming that and really shifting the mindset from type A personality, I have total control…[to] you have strengths, I have strengths. Let’s combine those, and let’s work on each other’s weaknesses.” – Assistant Principal PC

### Compared to the original theory of change, what local adaptations were made to the TPEG model and why?

To answer this question, we first present the findings within the four steps in a collaborative inquiry cycle: lesson planning, observation, feedback, and revision, as shown in Table 4. We note that while none of the three schools in this study continued to use TPEG in its original form, teachers at each site reported that they still incorporated steps of TPEG into their daily lesson planning.

**Table 4.** Summary of local adaptations to TPEG theory of action.

Collaborative inquiry step	Major finding
Lesson planning	Three collaborative styles were identified: planning together, sharing lessons, and sharing materials. Each style has its own strengths and weaknesses.
Observations	Peer observations were universally missing as part of the collaborative inquiry process. This is concerning because observations are vital for lesson revision.
Feedback	Teacher conversations during reflection varied from concentrating on emotional states to providing constructive professional support.
Lesson revision	Many teachers relied on their memories to collaboratively refine lessons the following year, though most agreed that those who updated lessons immediately after the reflection appeared to be more successful.

Lesson planning

We found that formally structured TPEGs faded in the schools after the first two years of implementation, and smaller collaborative groups that met daily or almost daily emerged. Some teams were required to use strict county curriculum standards and activities, so much of their collaborative time involved sensemaking to understand and organize the materials from their county or making minor changes to the prior year’s materials. Clark Middle teachers had an advantage when updating lessons because they had virtual access to materials from all teachers in the school. If a standard moved from one grade to another, which happened frequently, then Clark teachers could easily access the materials of the teachers who taught that standard previously. Many Clark teachers stayed close to the original lesson study model by collaboratively anticipating student questions and difficulties and how they might overcome them as instructors. Teachers at all three schools described building off each other’s experiences, pushing each other to try new techniques, and encouraging each other to see problems from new perspectives.

The collaborative teams overall had three preferred collaborative styles, each with different strengths and weaknesses. The first way of collaborating is what we call *planning together*, for which teachers meet to create identical or nearly identical lessons. This is the intended lesson study method of planning, and it has the distinct advantage that each lesson is created by a group of teachers who, if they communicate effectively and trust each other, can combine their knowledge and experiences to make an excellent lesson.

Another collaborative style is *sharing lessons*, for which teachers split their work into distinct units that are individually planned and prepared to share with their colleagues, who often do not change anything about that lesson before teaching it. For instance, one teacher described how each teacher on her team planned then shared with each other all the lessons for only one or two days per week. Each lesson is prepared individually, so the lessons mostly do not benefit from collaborative thought. However, it allows each teacher to have more time to devote to his/her portion of the lessons and other responsibilities, including those outside of work. Shared lessons improve the same way they would if the teachers were working alone while adequately storing and referencing materials year to year except that teachers involved in this method of collaboration have more time to spend on each lesson because they only have to work on a portion of the total lessons. One teacher described the process for her team:

“We actually set aside each day. Like I might have Tuesday/Thursday lessons, another teacher will have Monday/Wednesday lessons, and one other teacher will have the Friday lesson or a test that she creates. So, every week, I know I’ve got two lessons that I need to make, and they’re going to be awesome…If it’s an activity that requires worksheets or any kind of supplies, I make sure all of my colleagues have those things. So, all they have to do is show up and teach it.” – Teacher TC

The third style of collaborative lesson planning is what we call “sharing materials”. With this method of collaboration, teachers have a connection (virtual or in person) where they share ideas and techniques with each other that they are not expected to use. One Elwood Elementary teacher attributed her preference for this collaborative style due to her many years of experience teaching and her comfort with her own teaching style. She described this collaboration as a way to get new ideas instead of being boxed into another teacher’s style of teaching:

“I love to get copies of [my teammates’] notes. I love to just share what I’m doing with them, and if they don’t want to do it that way, that’s just fine with me…It’s not that I don’t want different ways or new ways. I just like to take the best of every aspect that I can find and then make it what I want it to be. Rather than everybody agree to say this and this and this and use this worksheet and do these notes on an active board. Some of that I love. But I don’t care for being, kind of, molded into this exact way of doing it…I probably sound like I just want to go off on my own with no collaboration and no teamwork, but that’s not the case at all. I do love to share and love to gain different ideas from other people, but I want to pick which ones I want to use and which ones I don’t.” – Teacher BE

Some teachers preferred this method of collaboration because it allowed them to hear new ideas without deviating from their preferred teaching style. Other teachers can *only* use this method due to staffing or structural constraints, such as not having a grade-subject collaborative partner. For instance, at Clark Middle School, the single science and single social studies teachers for the 7 ^th^ grade collaborated by discussing specific resources that would likely be effective for teaching either subject.

Observation

*To observe or not observe* was the most salient, discussed, and fretted about action step to the teachers in this study. None of the three schools sustained observations as part of the collaborative inquiry process. Many teachers became nervous when “tall people” came into their rooms or reflected that it was easy to try to put on a show when someone was observing. Teachers also struggled with exactly how they were supposed to conduct the observations. With Japanese lesson study and teaching-study groups in Shanghai, teachers are supposed to evaluate the lesson that was collaboratively planned, not evaluate the teacher him/her/themself. Some, but not all, teachers who had participated in TPEG seemed to understand that distinction, which helped teachers rationalize their way into accepting the observations. However, even those who understood the distinction had a difficult time making it work in practice. Most teachers understood that observations were times to focus on instructional practices, but they struggled to balance between keeping such focus and paying attention to student reactions, engagement, or work.

A few years after TPEG was introduced to Granville Elementary, the administration introduced the state practice called IPI to improve vertical alignment. With IPI at Granville, teachers were paired to observe each other teach and give feedback once each before they moved to another teacher. They did this process approximately twice per semester. The principal said it took about three years of mandatory IPI before teachers became excited about participating in it.

Another major obstacle for both TPEG and IPI observations was finding the time to do them. At each school, teachers of the same grade level shared a plan time, so they could only observe teachers at a different grade level if they were going to observe during that time. This was not a major problem for Clark teachers, who had 45 minutes of individual plan time every day. At Granville, however, plan time was more limited, so frequent observations significantly detracted from teachers’ tolerance of the practice. Administration provided substitutes for TPEG so teachers could observe each other when they otherwise would be teaching a class, though most teachers did not like leaving their students.

Teams at each school talked about or experimented with technology to ease the burden of observations. Granville teachers discussed videoing themselves teaching TPEG lessons, but they did not feel they had the equipment or expertise to do that well. At Elwood, teachers were able to figure out the technology, but they found it to be a “big load,” particularly with finding the time to watch the videos. There were also some teachers who felt uncomfortable being videoed. For all these reasons, virtual observations were not sustained at the three schools, either.

Feedback

Given that peer observations did not last in any school as part of the lesson planning process, the collaborative teams needed to find new ways of assessing whether students were engaged and learning the material, and how the lesson might be improved. At Clark Middle School, teachers evaluated lessons by paying attention to their own impressions of the lesson, including overheard student comments, and examining student assessment data. The method of remembering and recounting is practical, though it allows room for subjectivity and more importantly, limits the benefit of leveraging peer expertise. The principals noted, however, that with assessments that were standardized across classes, teachers were able to evaluate the strength of their lessons based on how well the students demonstrated their knowledge gain in class and through testing.

Elementary students were less able to express themselves and take frequent assessments, so Granville Elementary teachers had to find different ways of evaluating their lessons. Teachers reported watching students to see if they were “glazed out” or could correctly use new information later. Teachers tracked goals for their students, paid attention to teacher evaluations and their students’ standardized test score growth, and talked to teachers in the grade above to see if their students were adequately prepared. While these might help a teacher evaluate if s/he was a good teacher, most of these methods are not useful for evaluating individual lessons. Teachers used their recollections to debrief casually on many, but not all, individual lessons. Their critiques of the lessons were often based on whether the teacher liked the lesson and its delivery, rather than framing discussions specifically around how much they thought the students learned from it.

Collaborative teams at Clark Middle School, despite having more overall time dedicated to collaborative planning, stayed more focused during lesson reflections on whether and how their students learned and gave each other professional support. In one observed session, the collaborative team was examining the results of a quiz students had taken the prior week. The teachers compared how quickly their students completed the quiz and went over almost every question together. If there were discrepancies between classes, the teachers would compare exactly what they taught and how they taught it, bringing up particular comments or discussion questions that the collaborative group had not discussed before teaching. Other debriefing sessions were similar, with teachers comparing student assessment or assignment data, discussing questions that several students had gotten wrong, why they likely got them wrong, and how the teachers could adjust their next lesson to clarify misconceptions. Despite this level of detail, Clark collaborative teams rarely spent more than 10 minutes of their planning time debriefing on lessons.

Many of the teams at Granville relied on each other for emotional support during lesson reflection. In one collaborative session, teachers shared stories, usually funny or frustrating ones, from their classes. Some of these stories were to prompt a discussion about classroom management or teaching techniques, but many seemed to be about gaining emotional support. Little (1990) calls this storytelling and scanning for ideas, and she regards it primarily as a method for teachers to reveal their knowledge, intentions, and values to his/her peers and shape or reinforce their shared professional community. It is also what Little (1990) calls aid and assistance, where colleagues assist in the practice of teaching only when asked and avoid giving unwarranted advice on the stories. This was prevalent at Granville, where teachers often sympathized with the plight of their peers but only gave advice in moments when the teller was clearly seeking additional professional support.

Meanwhile, some teachers reported that feedback sessions could be nerve-wracking and unhelpful. Teachers could feel attacked when their lesson went poorly, or they might feel the need to keep information private or talk themselves up to colleagues to maintain their image as a competent teacher. One Elwood Elementary teacher exclaimed that she was able to use reflections to brag on her peers about what went well in the “model” lessons, and another felt it encouraged helpful self-reflection. However, others felt they had to give surface-level or biased feedback to avoid hurting people’s feelings. Some Granville Elementary teachers also felt this with their IPI observation feedback and expressed that it was not helpful to spend time on the IPI process if they could not give or receive substantive feedback. Based on Granville and Clark Middle, in particular, giving and receiving constructive feedback seems to be a skill that can be learned, so perhaps more time and training would alleviate potential concerns.

Lesson revision

Many Clark teachers reported that they always immediately updated lessons that needed a revision through a shared database. These changes were immediately helpful for the handful of teachers who taught the same lessons to different students from one day to the next, but many teachers did this simply for their own benefit in the following year. Teachers at the other schools reported reflecting verbally then trying to remember which lessons went well and which did not when lesson planning the following year.

While the four steps for the TPEG cycle provide the necessary structure to conduct disciplined collaborative inquiry, it is important to also examine the extent to which practices at the three schools strive to reach the essential objectives of building a professional knowledge base for teaching.

Public, deprivatized practice

Hiebert *et al.* (2002) emphasize that knowledge “must be created with the *intent* of public examination, with the goal of making it shareable among teachers, open for discussion, verification, and refutation or modification” (pg. 7). Teachers in this study who collaborated intended to share their lesson materials with each other, though those who collaborated *via* sharing materials did not open their creations up to be discussed, verified, or refuted by a group. Even if teachers rarely refuted lessons that were shared by colleagues, they were given the opportunity to do so and could discuss the lesson in depth after teaching it. Using this definition of public, the teachers who planned together or shared lessons were adequately making their teaching public.

Storing/sharing

With TPEG, lesson storage and sharing are supposed happen frequently throughout the process. Teachers pull lessons from storage when they are lesson planning, store lessons before teaching them, and store updated lessons after debriefing and revision. However, storage and sharing were not salient to many of the teachers in this study, and many only commented on them when prompted. Each school had different techniques for lesson storage and sharing. Teachers at Elwood had storage online that allowed them to share materials with each other and the principal for comments. At Clark Middle, teachers were required to use a central lesson repository for storing and sharing materials across the district. School administrators occasionally gave feedback on these stored lessons, and some teachers accessed other grades’ materials to stay informed about vertical alignment and to pull materials when standards changed grades. Teachers at Granville Elementary School often used localized storage techniques that varied by collaborative team. Lessons were usually kept on one teacher’s hard drive and/or in a filing cabinet. The Granville storage methods were therefore often used only as convenient “storage units,” rather than as extended spaces for collaboration.

Hiebert *et al.* (2002) assert that it is not enough to share locally with a few colleagues; professional knowledge must reach beyond the time and place they were created. Online county-wide repositories allow the lesson materials to reach more teachers than they otherwise would have. The rural county, where both Granville and Clark are located, had curriculum standards that were updated every few years, and teachers closely aligned their lessons with the standards. Perhaps this means that most lessons should only reach so far as the county. School systems that defer instead to other district, state, or even federal standards might want to extend their lesson storage system to those levels instead.

Mechanism for validation and improvement

At first glance, there is a mechanism for lesson validation and improvement embedded in the collaborative inquiry practices explored in this study. Teachers at Clark Middle, in particular, spent time together dissecting their own impressions and student assessment data to reflect on their teaching and improve lessons. The question becomes more complicated when considering whether the teachers could adequately reflect on their teaching, given that they did not observe each other teach the lessons. Lewis *et al.* (2006) consider live observations to be critical to lesson study as part of the research process.

Additionally, Hiebert *et al.* (2002) differentiate between local knowledge generated by the teachers themselves, which might not always be accurate, and expert knowledge or repeated evaluation in different contexts. Expert knowledge comes from instructional experts such as instructional coaches, some administrators, and researchers. At Clark Middle School, the instructional coach spent half the day teaching, and her collaborative partners expressed their appreciation of having her on their team to share expertise. All three schools had instructional coaches, but their roles were usually to provide assistance based on requests from the teachers. At Clark Middle and Granville Elementary, teachers had access to materials from other teachers in the county (repeated evaluation), but they mostly relied on their own team’s materials. Because of this, the teachers in these schools primarily relied on local knowledge, which means that they were not guaranteed to be appropriately validating and improving their lessons.

The theory of change behind lesson study is that teachers collaborate by examining and improving lessons together to make themselves higher quality teachers, so their students get better instruction. While teachers and principals from these three schools reported that their lessons and instruction were improving, more evidence is needed to see whether the teachers themselves were learning and improving. Imagine a situation in which two teachers, one novice and one veteran, are collaborating. The veteran teacher brings many years of experience to the partnership, and the novice teacher brings knowledge of new techniques and technologies. If neither teacher goes into depth about *why* their contributions are important or *how* to best incorporate them into future lessons, we will not know if their collaboration leads to improved instructional practices and contributes to a shared knowledge base.

This is not a hypothetical problem. Morris and Hiebert (2011) discuss the Japanese lesson study and emphasize that stored lesson plans include rationale for teaching decisions and changes, so that other teachers can later use the lessons in new contexts. One administrator in this study shared that she thought about this potential problem regarding her newer teachers, who had only ever known collaborative planning. She worried that they might leave her school and be unable to plan new high-quality lessons on their own.

Despite the intermediary, the ultimate goal for the collaborative inquiry models is to improve instruction. Teachers and principals stated that collaboration made them grow as teachers by allowing them to learn from their peers, forcing them to detail their thought processes when planning lessons, holding them accountable to do high-quality work, and decreasing total workload. The administration at Clark Middle School, though, expended effort to track concrete changes:

“One, we were a rewards school this year. Our overall student [testing] data has gone up…We have also seen [improvements] in teacher overall observation scores on the TEAM [evaluation] rubric… [The principal] gets evaluated every year just on his ability as an administrator, and he has seen a rise in his scores in this. We have seen a rise in happiness ratings [from approval surveys] from our teachers. And one thing that is always on the evaluation is, “Please don’t stop our collaborative plan.” – Administrator PC

This administrator, and teachers at both Clark Middle School and Granville Elementary School, attributed the increases in student test scores, teacher evaluations, the principal evaluation, and teacher approval ratings directly to their collaborative practices. Collaboration also allowed for teachers to participate in sensemaking and emotional support activities in what is usually an isolated career path.

## Conclusion

Our study finds that teachers in three Tennessee schools made strides in becoming collaborative partners to improve their teaching. We come away with five major action steps that support collaborative inquiry cycles at the school level: Form grade-subject collaborative teams; create time that is embedded within the school day for collaborative teams to meet; instruct and model how to productively collaborate, with particular emphasis on how to give constructive feedback; maintain high expectations for participation in collaborative planning (perhaps by making it mandatory) among both administration and the teachers themselves and for creating high quality lessons; and get buy-in through proof that collaborative inquiry cycles will be worth the commitment by observing other schools with successful collaborative practices and/or creating time for teachers to see changes in their own practices.

Our findings also identify four pathways that teachers and school administrators can take to implement or adapt the inquiry cycles into forms that are more successful and/or sustainable in their schools: Using collaborative inquiry to plan lessons multiple times per week, instead of spending multiple weeks examining one lesson; conducting peer observations in-person and scheduled so that teachers do not have to leave their students with a substitute teacher; conducting thorough, though not necessarily lengthy, reflections on every lesson; and using online spaces that are shared among the collaborative team, the administration, and preferably, teachers outside the collaborative team to store lesson plans and reflection notes instead of localized storage units.

Furthermore, our study identifies a few areas in which collaborative teams at these three schools struggled that future research should address. First, many teachers struggled to appropriately gather data to reflect on the success of their lessons. When they observed each other, they did not fully understand or internalize what exactly to observe, and the model teacher often felt uncomfortable in the process. When teachers did not observe each other, they used a variety of techniques to gather information about their lessons, but those sources were likely not as objective and meaningful as they could be. Second, it is still unclear how to move from local knowledge (from the teachers themselves) to incorporating expert knowledge to better validate and improve lessons. Principals or other administrators could participate in collaborative meetings as instructional experts, but the data show that it is often important for administrators to maintain distance from collaborative inquiry practices to ensure that teachers feel ownership over the process. One possible solution would be to embed instructional coaches, perhaps ones who also teach the same content, into teams so they are considered insiders. Other questions that this research opens up are about how to help schools transition from noncollaborative to highly collaborative environments. For instance, can collaborative teams share materials and/or lessons to ease the transition from individual planning to the intensive method of planning together?

This study is distinct from others in the literature because of its focus on decision making and tradeoffs when school administrators and teachers implement and adapt collaborative inquiry cycles in American schools. It helps fill some of the “critical research needs” that Lewis *et al.* (2006) identify. In particular, it describes a lesson study practice called TPEG and how it was supported and evolved over time in three schools, each with distinct characteristics and ways of practicing lessons study. It also helps explain the mechanism by which collaborative inquiry can improve instruction by improving lesson plans, which is why many teachers in this study decided to collaborate on every lesson instead of focusing on one lesson for several weeks at a time.

This research has several limitations. One is that the primary data source for this research is interviews, as respondents could misremember or miscommunicate activities, actions, and perceptions. Another limitation is the interruption of data collection. At Elwood Elementary, we wanted to do observations and complete more interviews, including ones with the former principal and instructional coach who were instrumental in introducing and supporting TPEG at Elwood. However, we were asked to cease data collection at Elwood after having completed only seven phone interviews due to an uptick in student disciplinary issues that the principal said kept the staff too busy to participate. Some of the plan sessions we observed at Granville Elementary and Clark Middle were apparently slightly more casual than usual sessions. Another major limitation is external validity. While we could identify and analyze patterns and processes in Elwood Elementary School, Granville Elementary School, and Clark Middle School, the findings may not directly translate to other schools. In particular, these were all middle- or high-achieving schools prior to the introduction of TPEG, and the interviews were only conducted during a limited period. We do expect, however, many of the decisions and thought processes around balancing resources and improving instruction will be consistent across many schools.

 “The American teaching culture, I think, is very different [from that in Shanghai] in the fact that teachers are not natural sharers. We have always really taught with a closed-door mindset” (Assistant Principal PC).

Despite decades of efforts in building professional communities, the teaching culture and organizational structure in American schools today present challenges to implementing and sustaining collaborative inquiry cycles as a method to improve student learning and teacher working conditions. By describing how three schools navigated the process of conducting collaborative inquiries, our study will help inform future efforts in supporting teacher professional learning and improving instructional practice in schools.

## Declaration of source work

The first draft of this work was published as a dissertation thesis ( Carr, 2020) and can be found in Vanderbilt University Institutional Repository.

## Ethics

This study was approved by the Behavioral Sciences Committee of the Institutional Review Board at Vanderbilt University (IRB #180759).

## Data Availability

Source data are not provided because the interviews cannot be effectively de-identified, and the IRB-approved consent forms promise confidentiality and that full interviews would not be released to outside parties. The interview protocols can be found using the following citation: Carr & Cravens (2022). Interview Guide for Teaching Without Boundaries: Interviews Exploring the Adaptation of Collaborative Inquiry to the American Context. Zenodo. https://doi.org/10.5281/zenodo.7422861

## Footnotes

^1^ ‘Elwood Elementary’, ‘Granville Elementary School’ and ‘Clark Middle School’ are pseudonyms used to protect participants’ identities. Any similarity with real school names is purely coincidental.

^2^ Interviews were conducted by phone at ‘Elwood Elementary’ as part of an extended pilot period, and the principal withdrew the school’s participation in the study before in-person interviews could be set up.

^3^ The letters after each quotation are confidential, unique identifiers for each respondent. The second letter is the school pseudonym: C (Clark), E (Elwood), and G (Granville); these schools’ pseudonyms are randomly generated and do not correspond to actual schools for the sake of protecting participants identities. The first letter is an alphabetical identifier based on the order of interviews at that school. So, "Teacher LC" was the 12th interview conducted at ‘Clark Middle School’.

## Author information

Olivia G. Carr is presently a consultant based at Education Resource Strategies; however, the research took place at Vanderbilt University in the Department of Leadership, Policy, and Organizations.

Xiu Cravens is a professor of the practice at Vanderbilt University.
