# Supplementary material for: Teaching without boundaries: interviews exploring the adaptation of collaborative inquiry to the American context
Source: Gates Open Res. 2023 May 15;7:70. [Version 1] doi: 10.12688/gatesopenres.14168.1 (PMC10285045; doi:10.12688/gatesopenres.14168.1)
Supplement: Peer review history

# Peer review: Reviewer response for version 1

**DOI:** 10.21956/gatesopenres.15474.r33350

**Published:** 2023-06-09

**Authors:** Haiyan Qian

**Affiliations:** 1 Department of Education Policy and Leadership, The Education University of Hong Kong, Tai Po, China

The study investigates teacher-led collaborative inquiry for instructional improvement using the TPEG model in the US, examining its local adoption and adaptation. It effectively contributes to instructional leadership literature by addressing the gap that collaborative inquiry is not commonly seen in the United States. The study is theoretically grounded through a thorough review of the collaborative inquiry cycles' construct and the TPEG model, and the theory of change. The research design is appropriate, with detailed information on the justification of sampling choice and data collection. I particularly appreciate the authors' clear explanation of the interview procedure and the description of the coding process.

 For data analysis, it would be ideal if the authors could make the final codebook available, including code names, definitions, and examples. The findings are clearly outlined to address the two raised research questions. They also seem to be blended with the Discussion. Lastly, the Conclusion effectively supports the findings, with defined study implications and some limitations elaborated.

 Overall, the paper is well-written and coherent in structure. It is a valuable piece of research work, particularly regarding the application of the TPEG model in different social and cultural contexts.

Is the work clearly and accurately presented and does it cite the current literature?

Yes

If applicable, is the statistical analysis and its interpretation appropriate?

Not applicable

Are all the source data underlying the results available to ensure full reproducibility?

No source data required

Is the study design appropriate and is the work technically sound?

Yes

Are the conclusions drawn adequately supported by the results?

Yes

Are sufficient details of methods and analysis provided to allow replication by others?

Yes

Reviewer Expertise:

Educational leadership and educational development in China

I confirm that I have read this submission and believe that I have an appropriate level of expertise to confirm that it is of an acceptable scientific standard.

# Peer review: Reviewer response for version 1

**DOI:** 10.21956/gatesopenres.15474.r33348

**Published:** 2023-06-02

**Authors:** Jianjun Wang

**Affiliations:** 1 Department of Education, East China Normal University, Shanghai, China

Over the past three decades, educational policy makers and school administrators are not only facing pressures from the gap between the ideals and reality of education, but also from so-called “learning gaps” or “teaching gaps” comparing with other countries. In response to these pressures, hundreds of even thousands of reform projects have been taken to improve the performance of teachers (who are regarded as the “active agents” of the education system). For such projects, one can always expect to see their "ultimate consequences", that is, their contribution to students’ academic achievements. We do have a lot of such kind of research papers published every year. However, since there are always a huge range of intervening variables between "how teachers are supported" and "how students perform", it should be logically questioned when examining their direct correlations, even that such an examination is technically feasible.

 In fact, there theoretically exists a more reliable method to examine the "efficiency" or “worthiness” of a reforming educational project: to examine the sustainability of the changes which the project advocates over a span of duration. If the measures of a reform project do not yield the desired effects, school administrators and teachers, as rational professionals, are unlikely to continuously adhere to a such a project. Thus, if the actions advocated by a project can be sustainable (with necessary and reasonable adaptations), especially when it requires a significant "re-construction" (such as re-organization of personnel, reschedule of work time), we can confidently say that the "intrinsic values" of these new actions are recognized (and realized) by administrators and teachers, although they may not be able to clearly define what these “intrinsic values” are.

 Olivia G. Carr and Xiu Cravens provides an excellent example of the above perspective in this paper. Teaching Excellence Peer Group (TEPG) is a project initiated by a team at Vanderbilt University a decade ago to change the way teachers engage in their professional activities, which requires significant re-definition and re-arrangement of the roles, relationships, and rules of school system.

 In 2013, I was very honored to contribute to the early stage of the planning and design of TEPG, by introducing what we usually do and what problems we were facing in teachers’ collaborative lesson planning, peer observation, feedback and revision in Shanghai. But from the very beginning I was very skeptical that such a project could “survive” in the United States: In a professional culture that places a long-standing emphasis on autonomy, it may be a “mission impossible” for TEPG to promote collaboration between peers, which is the cornerstone of this project.

 That is why I was so surprised and delighted when I read this paper. Although, as the authors have pointed out, the findings presented in this paper are subject to many limitations in terms of the source and nature of the data, and, they do not calculate out a multi-level correlation model to show how TEPG “raises” the scores of students, TEPG has successfully survived and thrived in these schools for a full decade! The sustainability of this project is by itself enough to illustrate its "intrinsic values", although it may take another decade for us to explore and define what these values exactly are. The second point that I am very pleased with is that, the findings of the paper show that all three case schools have made necessary adaptations of the original design of TEPG, which means, on a personal perspective, actions envisaged in TEPG tend to be "embedded" in these schools’ daily professional lives in a way that is more relevant to their context. Reading the findings of this paper concerning the “difficulties”,“obstacles”, or “problems” that schools or teachers encounter in TEPG project is even a more delighting job for me: during the past 25 years of working with schools and teachers across China, I find that those who are really engaging in collaborative inquiry (rather than just “coping”) are bound to encounter similar difficulties and troubles, and the deeper they go, the more obvious and tougher these difficulties become. It is a contrarian indicator: the uncover of difficulties and troubles shows that TEPG has been implemented.

 Next, I would like to comment on a few of the authors' main findings based on my own observations in China. The following comments, for the most parts, are more like an triangulation of the authors' findings:

    

1. Olivia G. Carr and Xiu Cravens describe the action steps which are actually adopted in three schools to promote peer collaborative inquiry: forming collaborative teams, scheduling collaboration, learning to collaborate, setting expectations for collaboration, and cultivating buy-in. This is very impressive since I find that in China, although there are differences among schools and at different stages of history, the "basic steps" that make collaboration between/among teachers possible are always stable. We tend to summarize them as 5Ts: Team, Time, Task of teamwork, Trust, and Take-away (enabling each member to “gain” something from every collaborative action). The findings of this paper suggests that the steps taken by the three Tennessee schools to promote teacher collaboration are generally rather the same, or at least similar, to the their Chinese counterparts. This reinforced my holdings that there is always something common in teachers' collaborative inquiry, regardless of the distinct cultures in which collaboration takes place.
2. Another of my observations adheres to the findings of Olivia G. Carr and Xiu Cravens is that, most of the problems and troubles we encounter in the process of promoting teacher collaborative inquiry can be attributed to the quality of one or more of the above “action steps”, and the most critical factor is always “Team (or teaming)”. For example, if a group of teachers form a team with shared objectives, responsibilities, resources, and high level of engagement, time becomes more of a trivial problem because team members can always squeeze out or even "create" time to collaborate; and similarly, in such a "real" team, trust and commitment are norms that need not active maintenance.
3. The authors report that schools have made adaptations upon the original (designed) model of TEPG. For instance, teachers have adopted three different strategies in collaborative lesson planning: planning together, sharing lessons, and sharing materials. Based on my observations, these different strategies (which are also common in China) indicate, again, that there exists a “universal pattern” that deserves more attention and effort onto research in the future.

Finally, I hope to remind the authors and readers of this paper, especially those who are interested in trying TEPGs in their own schools, to consider the following issues, which I have encountered in my research and promotion of teacher peer collaboration in China, and which are very likely to challenge the conducting of TEPGs in United States:

1. Scope of collaboration. The rapid development of mobile Internet technology in the past ten years has dramatically impacted, reshaped, and restructured teachers’ peer collaboration in China: When a “team” does not have to be confined to one school (or school district), how can we initiate, support, organize, manage, and evaluate “large-scale-teamwork” and at the same time optimize “local-teamwork”?
2. Clarity of criteria. During the past two decades in China, almost for every two or three years, the Education Authority will issue a new plan in reforming the curriculum, instruction, learning, or evaluation. Such a ridiculous “culture” makes the criteria for judging the quality of teaching and learning increasingly ambiguous, and causing a growing disagreement among teachers regarding how to decide “what counts as a good lesson”. Teacher peer collaboration is a process of pedagogical reasoning, decision making, and action. When there are no more shared criteria for judgement, how can peers co-planning, co-observing, and co-revising the lesson on hand?
3. Commonness and differentiation in collaboration. In the past, at least in China, we assumed that any individual teacher is now and in the future will encounter "same problems to be solved", which makes collaboration more valuable than individual endeavor (because it could provide solutions superior to individual struggles). In recent years, however, individual differences among teachers, not only in terms of their personal experience, knowledge bases, personalities, or teaching styles, but also in the professional problems they have to solve (for example, because of individual differences among pupils), becomes a common fact. If the professional duties, tasks and missions are inherently different among individual members of a group, what should teacher collaboration do to benefit each and every one who is involved?

Is the work clearly and accurately presented and does it cite the current literature?

Yes

If applicable, is the statistical analysis and its interpretation appropriate?

Not applicable

Are all the source data underlying the results available to ensure full reproducibility?

Yes

Is the study design appropriate and is the work technically sound?

Yes

Are the conclusions drawn adequately supported by the results?

Yes

Are sufficient details of methods and analysis provided to allow replication by others?

Yes

Reviewer Expertise:

teacher education, curriculum and instruction, university school collaboration

I confirm that I have read this submission and believe that I have an appropriate level of expertise to confirm that it is of an acceptable scientific standard.
